# Supplementary material for: A Microgel-Based Platform for Tunable Expansion and Function of γδ T-cells
Source: bioRxiv. 2026 Jul 24:2026.07.23.740340. Preprint. [Version 1] doi: 10.64898/2026.07.23.740340 (PMC13420452; doi:10.64898/2026.07.23.740340)
Supplement: Supplement 1 [file media-1.pdf]

Supplemental Information for

A Microgel-Based Platform for Tunable Expansion and Function of γδ T-cells

Authors
†,Favour Omafuvwe Obuseh [1,3], †,Junzhe Lou [2], Michelle Chang [2], Anqi Chen[2], David Weitz[2] *David Mooney[2,3]

Affiliations

1. Harvard-MIT Program in Health Sciences and Technology (HST), Cambridge, MA, USA
2. School of Engineering and Applied Sciences (SEAS), Harvard University, Cambridge, MA, USA
3. Wyss Institute for Biologically Inspired Engineering, Harvard University, Boston, MA, USA

†These authors contributed equally to this work

*Corresponding Author.
Email: David Mooney, mooneyd@seas.harvard.edu

This PDF file includes:

Figs. S1 to S4
Tables S1

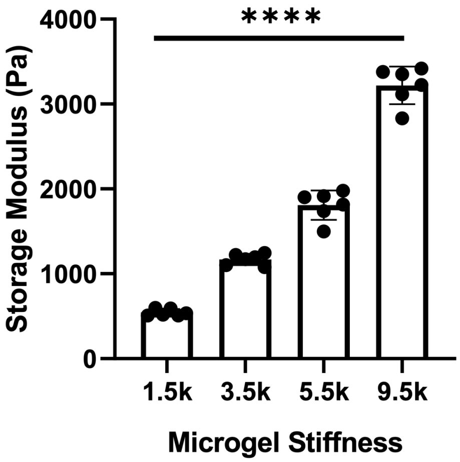

Fig. S1. Rheological characterization of microgels with different stiffness

Elastic modulus of microgels can be modulated by varying the degree of substitution of norbornene and tetrazine groups on alginate polymers, n=5 technical replicates. Statistics were performed using ordinary one-way ANOVA with Tukey's multiple comparisons test. $^{*}P < 0.05$, $^{**}P < 0.01$, $^{***}P < 0.001$, $^{****}P < 0.0001$; ns, not significant.

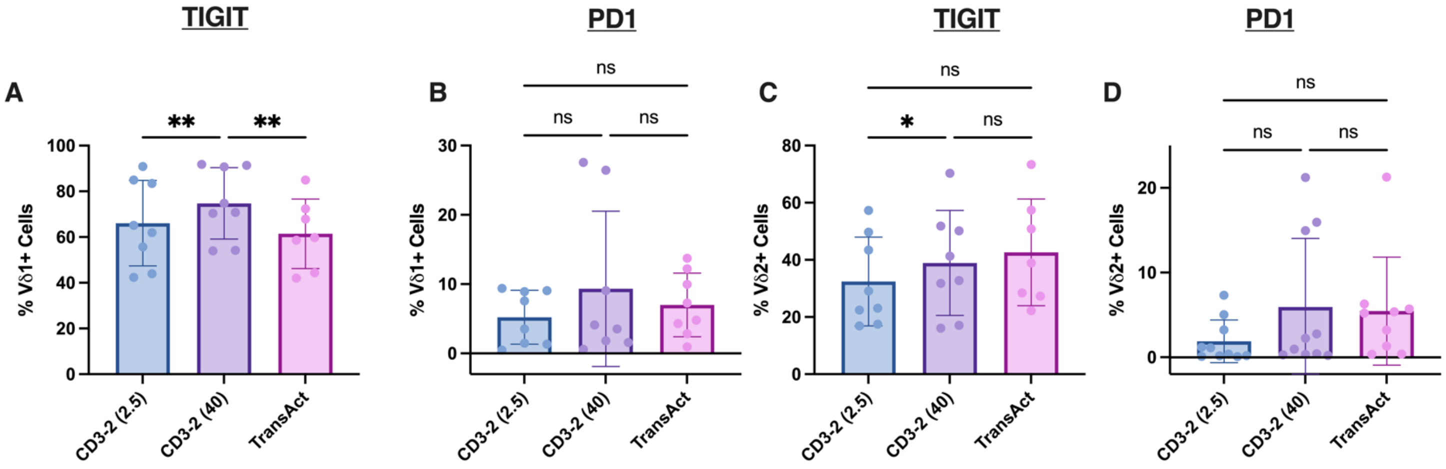

Fig. S2. TIGIT and PD1 percentages are modulated by stimulation density

(A-D) TIGT and PD1 Geometric mean fluorescence intensity for Vδ1 and Vδ2 cells respectively, across activation conditions. n=8 donors. Statistics were performed using ordinary one-way ANOVA with Tukey's multiple comparisons test. *P < 0.05, **P < 0.01, ***P < 0.001, ****P < 0.0001; ns, not significant.

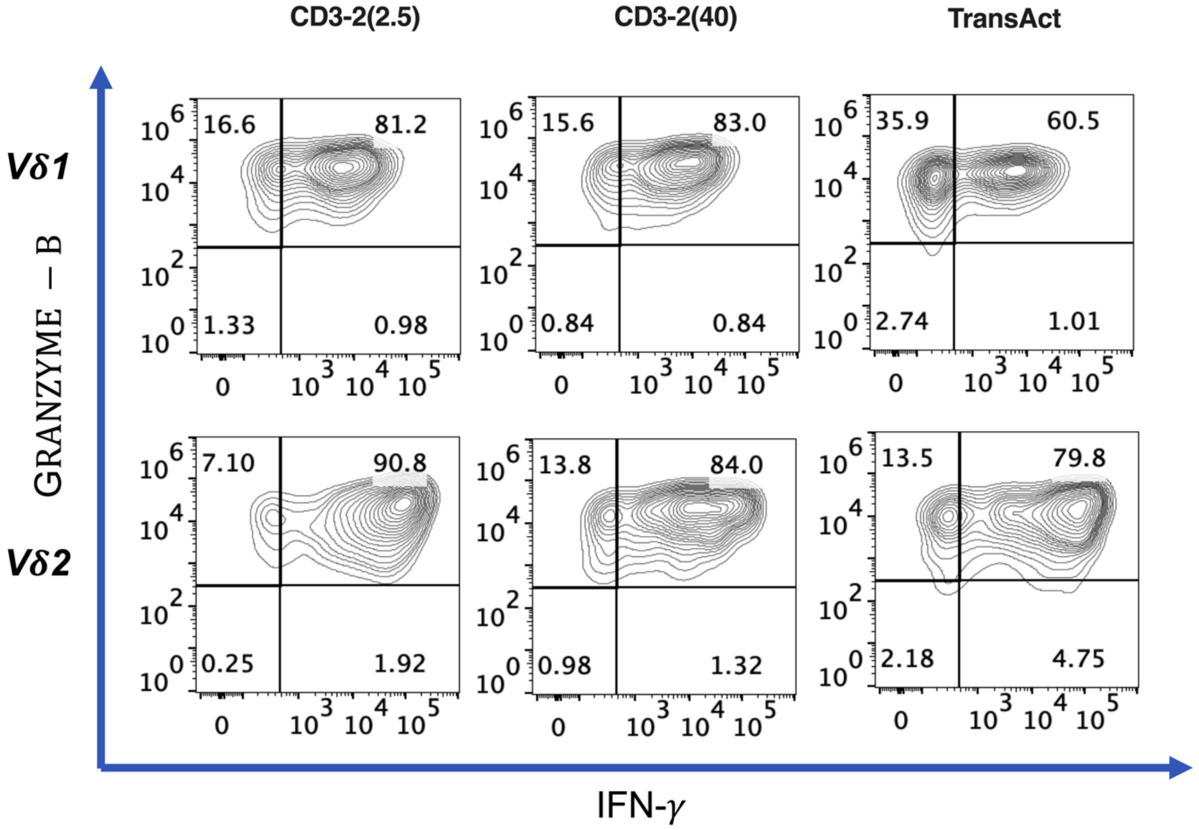

Fig. S3. Effects of ligand density on cytotoxic potential

Representative flow cytometry contour plots of Granzyme-B versus IFN-γ in expanded Vδ1 (top row) and Vδ2 (bottom row) γδ T-cells following culture on αCD3 functionalized microgels with αCD2 co-stimulatory molecule.

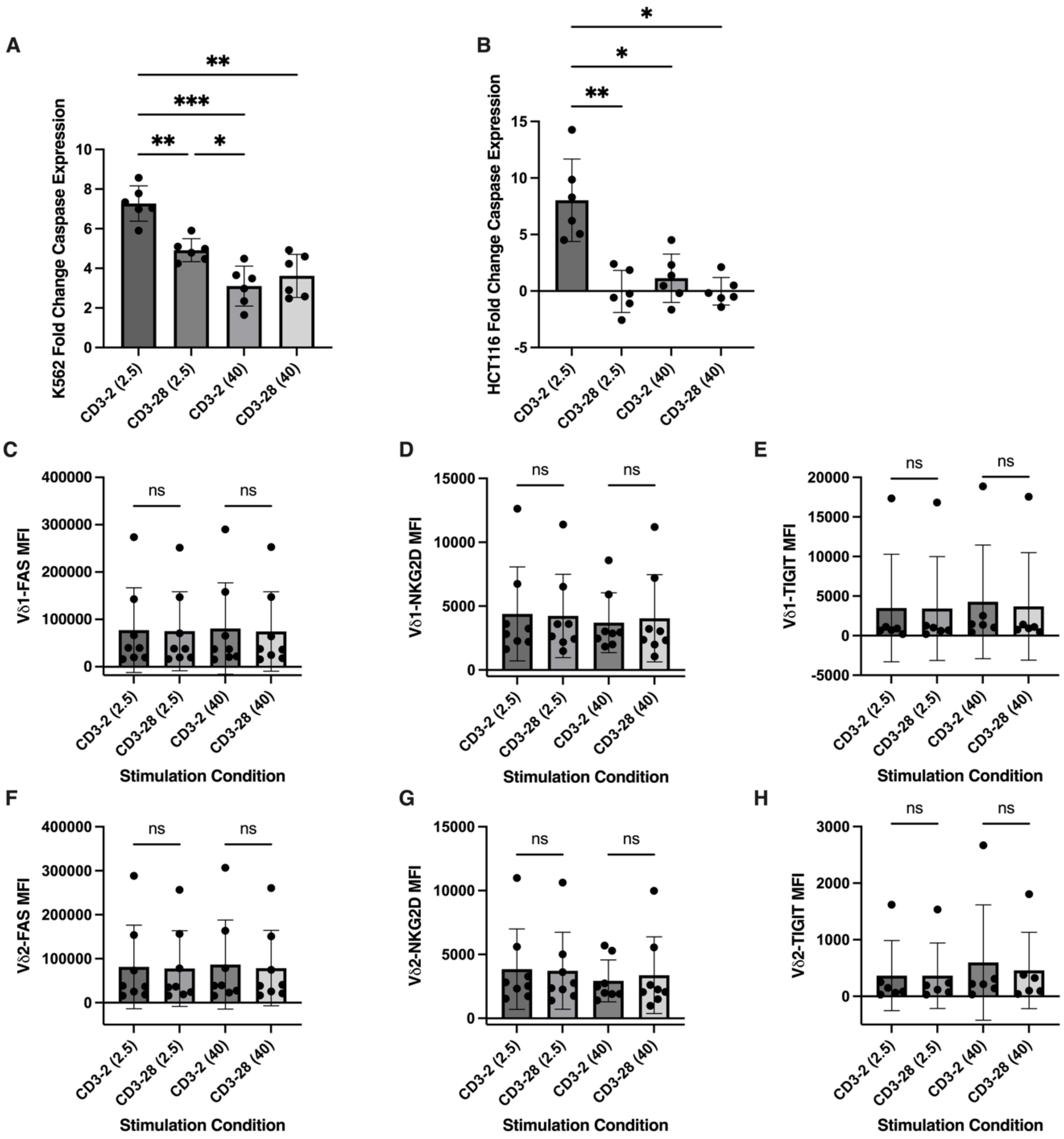

Fig. S4. Effects of CD28 co-stimulation on activation and cytotoxicity
(**A-B**) Fold change in caspase-3/7 signal in K562 (**A**) and HCT116 (**B**) target cells following 4 h co-culture with γδ T-cells expanded under the indicated conditions. (**C–E**) FAS, NKG2D, TIGIT geometric mean fluorescence intensity (gMFI), respectively, within the Vδ1 compartment across expansion conditions. (**F–H**) FAS, NKG2D, TIGIT geometric mean fluorescence intensity (gMFI), respectively, within the Vδ2 compartment across expansion conditions. For panels A–B n = 6 technical replicates; for panels C-D, F–G, n = 8 donors. E,H, n= 6 donors. Bar graphs show mean ± s.d. Statistics were performed using ordinary one-way ANOVA with Tukey's multiple comparisons test. *P < 0.05, **P < 0.01, ***P < 0.001, ****P < 0.0001; ns, not significant.

Staining Antibody	Manufacturer	Cat#
CD3 (RB705)	BD Biosciences	570237
TCR Vδ1 (PE)	Thermo Fisher	12-5679-42
TCR Vδ2 (APC)	BioLegend	331418
CD27 (BUV395)	Thermo Fisher	363027942
CD45RA (BV510)	BioLegend	304142
PD-1 (BV605)	BioLegend	367426
NKG2D (PE-CY7)	Thermo Fisher	25-5878-42
DNAM-1 (BV785)	BioLegend	338322
NKp30 (BV711)	BioLegend	325218
CD95/FAS (PE-CY5)	Thermo Fisher	15095942
Live/Dead NIR viability dye	Thermo Fisher	L10119
TIGIT (BV421)	BioLegend	372710
TCRγδ (PE/Dazzle)	BD Biosciences	331226
IFNγ (FITC)	BD Biosciences	554551
Granzyme B (PE-Texas Red)	Invitrogen	GRB17
TNFα (BV421)	Thermo Fisher	404-7349-42

Table S1. Antibodies used for experiments
List of antibodies used for flow cytometry, with manufacture's catalog number provided. The antibodies were used at 1:60 dilution, except for Live Dead NIR which was 1:1000 dilution.
